# Changes in prevalence of calcaneal spurs in men & women: a random population from a trauma clinic

**DOI:** 10.1186/1471-2474-15-87

**Published:** 2014-03-15

**Authors:** Hechmi Toumi, Ryan Davies, Marija Mazor, Raphael Coursier, Thomas M Best, Rachid Jennane, Eric Lespessailles

**Affiliations:** 1 EA4708 Orleans University, IPROS, CHRO, 1, rue Porte-Madeleine, BP 2439, Orleans cedex 1 45032, France; 2 Princess of Wales Hospital, Coity road, Bridgend, Wales CF31 1RQ, UK; 3 Groupement des Hôpitaux de l’Institut Catholique de Lille (GHICL)/Faculté Libre de Médecine, F-59000 Lille, France; 4 Département de traumatologie-orthopédie France, UC Lille, Lille, France; 5 Division of Sports Medicine, Department of Family Medicine, Sports Health And Performance Institute, The Ohio State University, Columbus, OH 43221, USA

**Keywords:** Achilles, Plantar, Spur, Men, Women

## Abstract

**Background:**

This study reports the changing prevalence of ankle (Achilles and plantar) spurs with age, in order to comment on their significance to rheumatologists.

**Methods:**

1080 lateral ankle radiographs from each of 9 (50 men and 50 women) age cohorts from 2 to 96 years old of patients attending a trauma clinic were examined and spurs classified as small or large.

**Results:**

The prevalence of both Achilles and plantar spurs in relation to the age categories and sex was variable. Overall, there was 38% of the population who had a spur (Achilles or plantar) and only third (11%) with spurs at both sites (Achilles and plantar). Large spurs were more prevalent in older individuals (40 to 79 years). There were no large plantar spurs in individuals <40 years of age and only 2% for the Achilles. The prevalence of spurs (Achilles and plantar) was significantly higher for woman than men in individuals <50 years of age. There was a notable moderate positive correlation (r = 0.71) between both plantar and Achilles spurs for women <30 years of age but no correlation for men (r = -0.03).

**Conclusion:**

Plantar and Achilles spurs are highly prevalent in older people and the radiographic appearance of spurs differs between men and women. In individuals < 50 years of age, spur (Achilles and plantar) formation is more common in women than in men. Additionally, there was a notable moderate positive correlation between Achilles and plantar spurs for women <30 years of age.

## Significance and innovation

• Plantar and Achilles spurs are highly prevalent in older people and the age of onset of spurs differs between men and women.

• Individuals < 50 years of age, spur (Achilles and plantar) formation is more strikingly common in women than in men.

• There was a notable moderate positive correlation between Achilles and plantar spurs for women <30 years of age but not for males.

## Background

Many patients with foot pain have often been diagnosed with “heel spurs.” Such a diagnosis resulted from an x-ray that revealed some extra bone where the plantar fascia attaches to the calcaneus. This extra bone is also called a “spur.” However, the pathophysiology of plantar spurs remains controversial. According to Bergmann [1], it originates from the repetitive traction of the insertion of the plantar fascia into the calcaneus, which leads to inflammation, and reactive ossification of the enthesis. However, Kumai and Benjamin [2] have challenged such a belief proposing that plantar spurs develop from vertical compression instead and cannot be traction spurs, as they do not develop within the plantar fascia itself. They are thus fundamentally different from spurs in the Achilles tendon since they develop as a consequence of degenerative changes that occur in the plantar fascia enthesis. Such an explanation is consistent with several studies, which have found that the bony trabeculae of plantar spurs are vertically oriented; suggesting that the stresses responsible for spur formation may be the result of vertical loading [3]. Recently, similar findings by Weiss [4] showed that dorsal spurs are in part the result of sustained activities, but plantar spurs result from long periods of standing and excess weight.

Although spur formation in the Achilles tendon and the plantar fascia is a well-recognised condition, the connection between these spurs and their prevalence with age and sex remain debatable [5]. In the only three large-scale epidemiological studies of Achilles and plantar spurs there are unequal numbers of subjects in the different age cohorts and a lack of adequate representation of older individuals [3,6,7]. Moreover, more women than men are typically recruited in their studies. Furthermore, size of the spur has not been adequately addressed [3,6,7]. This information is important in understanding the natural history spur formation and/or regression. To begin to address these limitations, Menz et al. [5] evaluated the prevalence and correlation of plantar spurs in a large sample of older people. While important, this study examined only subjects aged 62 to 94 years and was not a random sample of clinic patients. Hence the findings may not be generalizable to the broader community.

The purpose of the present investigation is to analyse the size and prevalence of both enthesophytes in the Achilles tendon and plantar spurs in the general population, in order to establish reliable base-line information that is useful for evaluating the pathological significance of spurs in rheumatic disorders. We have based the study on data from a large population of patients (1080 subjects) who attended the trauma clinic at a local hospital between June 1st 2005 and January 21st 2006, and ensured that equal numbers of individuals (men and woman) were included in each of the 9 different age cohorts examined. It is the first in a series of investigations aimed at improving our understanding of age related formation and regression of Achilles and plantar spurs.

## Methods

**Figure 1:**
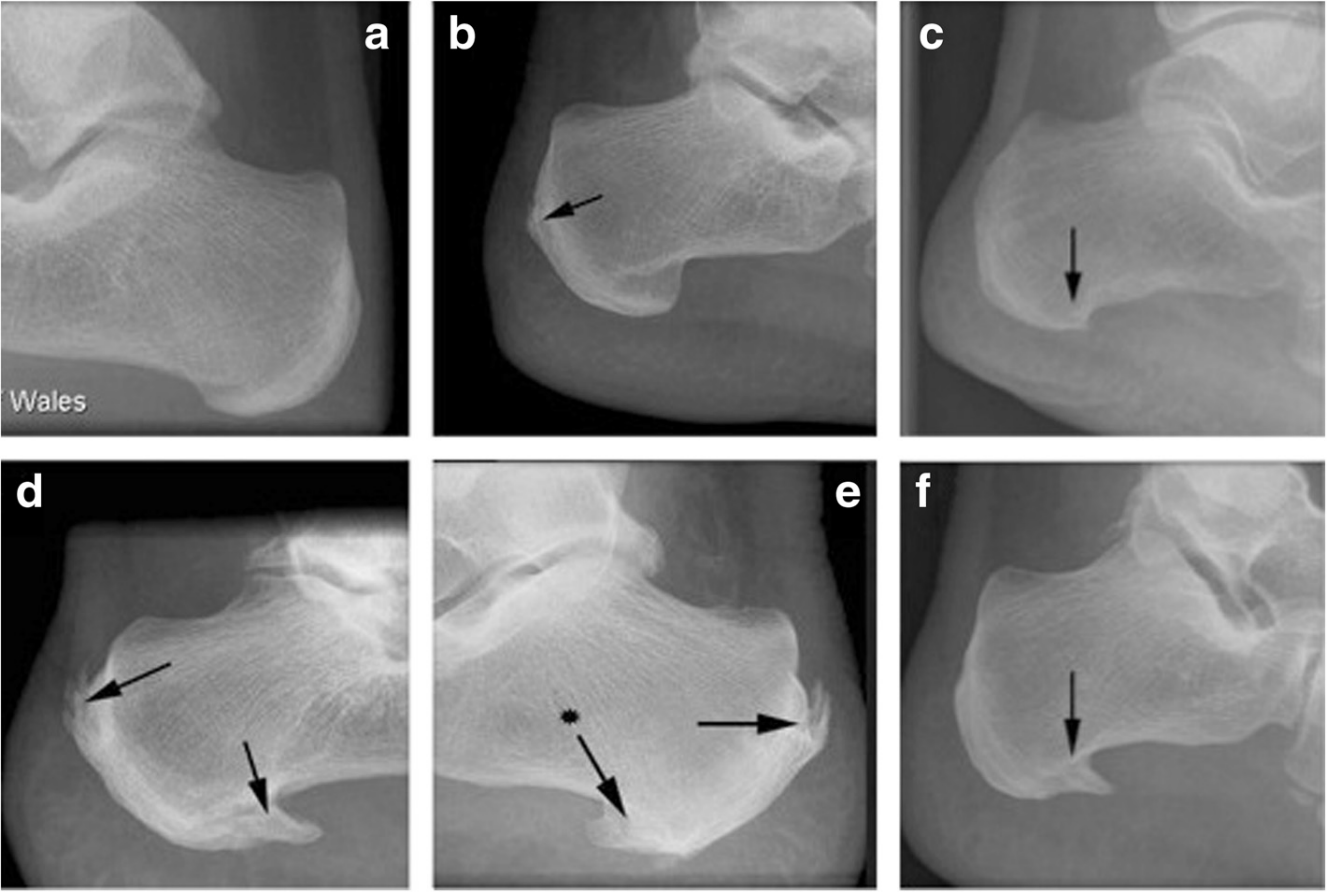
**Example of small and large spurs used to grade the sizes of spurs (arrows). (a)** no spur, **(b)** small Achilles spur, **(c)** small plantar spur, **(d-e-f)** large Achilles and plantar spurs.

The study was conducted in accordance with the Declaration of Helsinki and approved by a board institution. All patients where anonymous, no consent was needed and an ethics committee (South West Wales Research Ethics Committee) in accordance was obtained (agreement n° GH/KCC49 contract-30th of April 2009). A total of 1080 lateral view ankle radiographs on patients (ages 2-96), taken between June 1st 2005 and January 21st 2006 in the trauma clinic of the University Hospital of Wales, Cardiff were randomly selected and examined. Radiographs were taken for foot related disorders by the same medical imaging department using the same procedure. In order to ensure anonymity, patient names were not recorded. The ethnicity of the patients was unknown (because of the anonymous nature of the survey), but the 2001 national consensus suggests that the majority of patients visiting the clinic were likely to have been Caucasian. Note that Wales population-Caucasian 97.9%/mixed 0.6%/asian 0.9%/black 0.2%,/chinese 0.2%/other 0.2%. ‘2001 Census of population, Office for national statistics. In order to select the patients randomly, a search patient generator was used to select 100 radiographs (50 **men** and 50 **women**) from each age group, in order to permit an objective comparison as a function of age. This was chosen because 50 was the lowest common denominator of radiographs available. Nine groups were therefore analyzed; 0-9 years, 10-19 years, 20-29 years, 30-39 years, 40-49 years, 50-59 years, 60-69 years, 70-79 years, and >80 years. Where bilateral radiographs were available, one foot only was randomly selected for analysis. A spur was considered large when there was a prominent peak or peak with sub-structure present, a small spur being any alteration to normal surface contour of the calcaneus at the Achilles/plantar insertion. Examples of the x-rays obtained in the study are shown in Figure 1. In order to determine the reproducibility of the scoring system, particularly the dissimilarity between small incompletely bridging to large complete bridging, a pilot survey was conducted of 50 radiographs. The primary examiner and three other independent observers blindly viewed the radiographs and recorded scores for each image (i.e. none = 0, small = 1 and large = 2). The differences between the scores recorded by the 4 individuals were non significant (weighted kappa test, value =0.91) indicating that the scoring system was reliable for a single observer. Thus, all radiographs were subsequently scored by one examiner (second author). Due to their irregular shape no attempt was made to directly measure the length of the spurs or any delineation between spurs located in the plantar fascia and those located in the intrinsic musculature.

### Statistical analysis

The number of spurs of each type that was reported for each age group was expressed as a percentage of the total number of subjects in the group. The Kolmogorov-Smirnov test was used to assess data normality. Where necessary, a non-parametric statistical test (chi-square) was performed to compare the different variables measured and the significance was accepted at p = 0.05. With regards to the correlation between the different size of spurs, age and sex, a Spearman test was used. The correlations were considered very strong when the r value was above 0.8, moderate between 0.60–0.79, fair between 0.30–0.59 and weak when below 0.29 [8].

## Results

**Figure 2:**
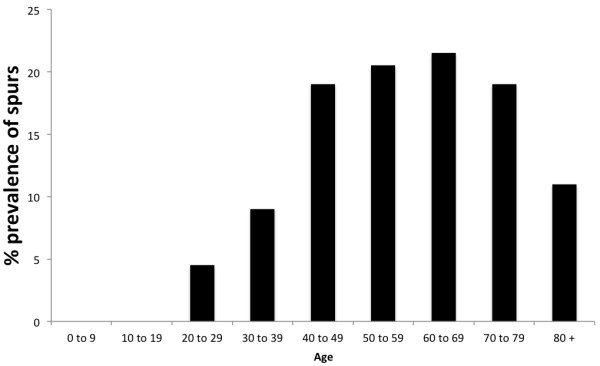
**The prevalence of individuals with both an Achilles and plantar spur in different age cohorts.** Note that the prevalence of individuals with both spurs increased up to 69 years of age and decreased thereafter.

**Figure 3:**
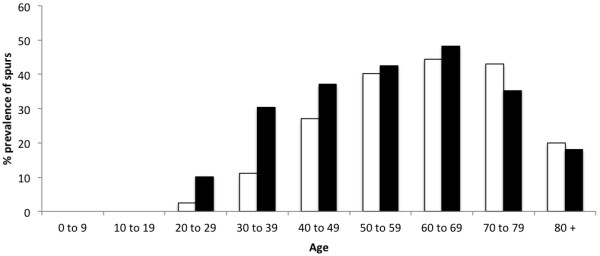
**The prevalence of plantar spurs in men and women by age category.** Note that the prevalence of plantar spurs were significantly higher for women (black bars) than men (white bars) in individuals <49 years of age. In both sex spurs were first seen in 20–29 years old.

**Figure 4:**
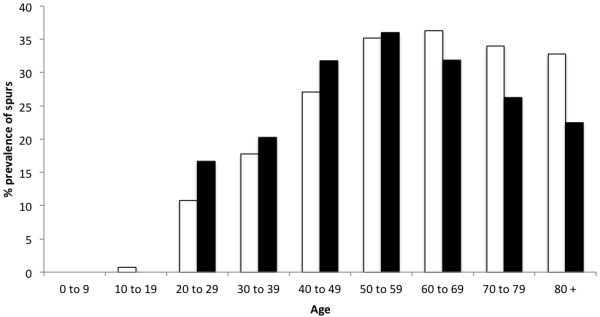
**The prevalence of Achilles spurs in men and women by age category.** Note that the prevalence of Achilles spurs were significantly higher for women (black bars) than men (white bars) in individuals <49 years of age. However, Achilles spurs were more common in men than women after 60 years old.

Typical examples of the different classes of spurs are shown in Figure 1. The overall prevalence of spurs (Achilles or plantar) in the population for all ages and both sexes was 38%. Conversely, the overall prevalence of individuals with spurs at both sites (Achilles and plantar) was only 11%. Plantar spurs were more common than Achilles spurs in women (p = 0.03) but not in men (p = 0.07) and their prevalence varied with age and sex (Table 1, Figures 2, 3 and 4). There were no spurs in individuals < 20 years of age. The prevalence of spurs peaked at 22% in the 60–69 year age group, but decreased thereafter (Figure 2).

**Table 1:** The prevalence of Achilles and plantar spurs (large and small) in men and women by age category (expressed in%)

	**Plantar**				**Achilles**			
	**Male**		**Female**		**Male**		**Female**	
	**Small**	**Large**	**Small**	**Large**	**Small**	**Large**	**Small**	**Large**
0 to 9	0	0	0	0	0	0	0	0
10 to 19	0	0	0	0	0	0	0	0
20 to 29	3	0	13	0	10	0	18	0
30 to 39	11	0	32	0	26	3	18	2
40 to 49	30	2	39	6	18	9	31	1
50 to 59	38	3	34	5	18	18	23	5
60 to 69	36	8	50	10	28	12	29	17
70 to 79	45	20	39	9	30	15	37	13
80 +	17	8	13	0	45	0	37	0

Although, there were no significant differences in the overall prevalence of spurs between men (41%) and women (38%) when the entire population was compared, when individual age cohorts and spurs of different sizes and location were considered, the pattern was far more complex. Plantar spurs were significantly more common for women <49 years of age than in men. Furthermore, there was a moderate positive correlation between plantar and Achilles spurs (r = 0.71; p = 0.03) for woman <30 years of age no correlation for men (r = -0.03; p = 0.01). The results are summarized in Figures 1, 2, 3 and 4, Table 1 and the most significant findings are detailed below.

### Plantar spurs (Figures 2, 3 and 4, Table 1)

There were no plantar spurs in individuals (men and woman) <20 years of age and only small spurs in 6% of individual aged 20–29 years. The prevalence of small plantar spurs continued to increase up to and including the 60–69 age group (where it peaked at 45%), but small spurs then became less common in the older age groups (26%). There were no large spurs in individuals <40 years of age and only 4% of individuals aged 40–49 years. Large spurs continued to increase up to and including the 70–79 age group (where it peaked at 14%) and then became less common in the older age group.

### Achilles spurs (Figures 2, 3 and 4, Table 1)

There were no Achilles spurs in individuals (**men** and **woman**) <10 years of age and only one in individual aged 20–29 years. The prevalence of small Achilles spurs continued to increase up to and including the 60–69 age group (where it peaked at 36%), but small spurs then became less common in the older age groups (22%). There were no large spurs in individuals <40 years of age and only 2% of individuals aged 40–49 years. Large spurs continued to increase up to and including the 70–79 age group (where it peaked at 20%) and then become less common in the older age group.

### Men versus woman and Achilles spurs versus plantar spurs (Figures 2, 3 and 4, Table 1)

Overall, plantar spurs were significantly more common in **women** than in **men** (p = 0.04). In both sexes, spurs were again first seen in 20–29 year olds except they were more frequent in **women** than **men**. Values were 3% versus 13% for the plantar spurs and 10% versus 18% for the Achilles spurs for **men** and **women** respectively.

### Correlation between plantar and Achilles spurs (Table 2)

**Table 2:** Correlation between prevalence of Achilles and plantar spurs in men and women with increasing age

**Age**	**Male**		**Female**	
	**r**	**P**	**r**	**P**
0 to 9	0.00	0.02	0.00	0.04
10 to 19	0.00	0.04	0.00	0.01
20 to 29	-0.03	0.02	0.71	0.01
30 to 39	0.15	0.01	0.51	0.03
40 to 49	0.33	0.04	0.43	0.04
50 to 59	0.38	0.01	0.38	0.02
60 to 69	0.36	0.02	0.36	0.01
70 to 79	0.35	0.00	0.36	0.00
80 +	0.29	0.02	0.34	0.01

Note there is a moderate positive correlation (r = 0.71) for women <30 years of age and fair >30 years old (0.30 < r > 0.59). Yet, in men there was only a weak positive correlation up to 40 years old (r < 0.15) and fair correlation in those >50 years old (0.30 < r < 0.38).

Spearman’s test applied to all age groups showed a fair positive correlation between plantar and Achilles spurs for women (r = 0.30) and weak positive correlation for men (r = 0.20). However, when applied to each category of age separately, we found a notable moderate positive correlation (r = 0.71) for women <30 years of age and fair >30 years old (0.30 < r > 0.59). Yet, in men there was only a weak positive correlation up to 50 years old (r < 0.29) and fair >50 years old (0.30 < r > 0.59).

## Discussion

Although from an anatomical perspective, spur formation in the Achilles tendon and the plantar fascia could possibly be related to the transfer of mechanical stress from site to site, the connection in the development between these spurs and their prevalence for age and sex remain uncertain. Overall, our findings confirm a variation in the prevalence of both Achilles and plantar spurs in relation to both age and sex. There were no large plantar spurs in individuals <40 years of age. The prevalence of spurs (Achilles and plantar) was significantly higher for women than men in individuals <50 years of age. In addition, there was a notable moderate positive correlation for women <30 years of age but not for men. It should be noted that this is the first epidemiological study of calcaneal spurs in which a high number of individuals was evaluated (1080 individuals) with equal numbers (100 individuals) assessed across nine different age groups.

Perhaps one of the most significant findings from a rheumatological perspective is that large plantar and Achilles spurs were absent in both males and females <40 years of age, similarly only 2% for spurs in the Achilles for the same age group. We thus suggest that if a large plantar spur or Achilles is present in a young male patient, a rheumatic disorder should be considered including degenerative or traumatic or inflammatory related enthesopathies. Furthermore, the low incidence of any type or size (small and large) of calcaneal spur (Achilles or plantar) in the <30 year group suggests that spurs take long time to build up. This probably because the enthesis organ dissipates the stress away from a bony insertion, this can explain why pathologic changes are seen adjacent to entheses as well as at them [9]. One more possible explanation could be that the development of such spurs is not predominantly caused by mechanical stress associated with exercise [10-12], as this age cohort is the most physically active. Although the absence of either type of calcaneal spur in individuals <20 years of age is intriguing (as this is the age when bone growth in general is most evident), it parallels the common clinical observation that osteophytes are rarely a feature of degenerative joint disease in young individuals [13].

The development of spurs differs between men and women. In individuals < 50 years of age, spur (Achilles and plantar) formation is more strikingly common in women than it is in men. However, this observation was not the case in individuals 50 years of age or older. The difference in individuals < 50 years could relate to shoe-wear, as surveys have shown that 37–69% of women wear high-heeled shoes on a daily basis [14] and increasing heel height is known to increase impact at heel strike [15] - thus altering foot biomechanics. Similarly, there was a notable moderate positive correlation between plantar and Achilles spurs (r = 0.71) for women <30 years. This is in line with previous reports [5] and supports the theory that in young women (<30 years old), Achilles and plantar spurs could be an adaptive response to longitudinal traction at the calcaneal enthesis, which induce an adaptive response in the heel/plantar fascia and visa versa.

Although the overall prevalence of spurs in women (Achilles or plantar) continued to increase with age and plantar spurs were more common than Achilles, it is interesting that the correlation between Achilles and plantar spurs declined after 30 years old. These findings suggest to us that plantar spurs could initiate from a different origin other than the longitudinal traction at the calcaneal enthesis. It could be an adaptive response to vertical compression of the heel rather than longitudinal traction at the calcaneal enthesis, which may have implications for the management of chronic heel pain in people >30 years old. A recent histological study has indicated that the bony trabeculae of spurs are vertically oriented, suggesting that the stresses responsible for spurs formation may be the result of vertical loading [3].

Both plantar and Achilles spur presence peaked around 70 years of age for women. However, in the men population both spurs continued to increase after age 70.

## Conclusion

Plantar and Achilles spurs are highly prevalent in older people and the development of spur differs between men and women. In individuals < 50 years of age, spur (Achilles and plantar) formation is more strikingly common in women than it is in men. Additionally, there was a notable moderate positive correlation between Achilles and plantar spurs (r = 0.71) for women <30 years of age. A future study has been planned to collect patient’s clinical findings and symptoms in correlation with spurs prevalence and progression. Note also that spurs increase with age therefore, it will be important to perform a statistical analyses between groups.

## Competing interests

The authors declare that they have no competing interests.

## Authors’ contributions

All authors contributed in the design, the analysis and writing the manuscript. All authors read and approved the final manuscript.

## Pre-publication history

The pre-publication history for this paper can be accessed here:

http://www.biomedcentral.com/1471-2474/15/87/prepub
